# Exercise training complementary to specialised early intervention in patients with first-episode psychosis: a feasibility randomised trial

**DOI:** 10.1186/s40814-021-00900-5

**Published:** 2021-08-19

**Authors:** Julie Midtgaard, Helle Schnor, Eik D. Bjerre, Tobias Jespersen, Nina Jelsøe, Nanna Frølund, Søren Seier, Jacob W. Rønbøg, Nikolai B. Nordsborg, Bjørn H. Ebdrup

**Affiliations:** 1 Mental Health Centre Glostrup, University of Copenhagen, Nordstjernevej 41, DK-2600 Glostrup, Denmark; 2 Department of Clinical Medicine, University of Copenhagen, Blegdamsvej 3B, DK-2200 Copenhagen N, Denmark; 3 The University Hospitals Centre for Health Research, Copenhagen University Hospital – Rigshospitalet, Department 9701, Blegdamsvej 9, DK-2100 Copenhagen Ø, Denmark; 4 University College Copenhagen, Tagensvej 86, DK-2200 Copenhagen N, Denmark; 5 Department of Nutrition, Exercise and Sports, University of Copenhagen, Nørre Alle 51, DK-2200 Copenhagen N, Denmark; 6 Center for Neuropsychiatric Schizophrenia Research (CNSR) and Center for Clinical Intervention and Neuropsychiatric Schizophrenia Research (CINS), Mental Health Centre Glostrup, University of Copenhagen, Nordstjernevej 41, DK-2600 Glostrup, Denmark

**Keywords:** Exercise training, Feasibility, First-episode psychosis, Randomised trial

## Abstract

**Background:**

The aim of this study was to examine feasibility of trial processes and group-based, structured exercise training in patients with first-episode psychosis.

**Methods:**

Twenty-five patients with first-episode psychosis took part in a two-arm randomised feasibility trial. They were individually randomised (1:1) via a computer-generated randomisation sequence and allocated to either an exercise intervention group (INT) or a control group (CON). Patients allocated to INT completed a physical exercise training programme at moderate-to-vigorous intensity, 1 h three times weekly for 8 weeks. CON patients were encouraged to continue their usual level of activity and were offered the training programme after 8 weeks. Primary outcomes included screening rate, recruitment rate, retention rate, attendance and adverse events. Secondary outcomes included heart rate response during training, cardiovascular health (VO_2max_, resting heart rate, blood pressure), body composition (muscle mass, fat percentage), muscle strength (sit-to-stand, grip strength, jump height) and balance.

**Results:**

Recruitment lasted 6 weeks and 86 out of 324 patients (27%) were screened, 71 of whom (83%) were deemed eligible. Twenty-five (35%) accepted inclusion (mean age 25.5; mean body mass index 25.1) and were subsequently randomised (INT = 13, CON = 12). Retention of patients was 76% and 52% at the 8-week and 16-week follow-up, respectively. Attendance was 43% (min. 9%, max. 96%). No significant changes were observed between groups in secondary physiological outcome measures.

**Conclusions:**

Feasibility was challenged by limited recruitment and retention rates, suggesting that modifications are required if a large-scale randomised controlled trial is to be conducted. Recommendations for modifications are presented and discussed.

**Trial registration:**

Clinicaltrials.gov, NCT03409393. Retrospectively registered.

**Supplementary Information:**

The online version contains supplementary material available at 10.1186/s40814-021-00900-5.

## Key messages regarding feasibility

- While some previous studies have explored exercise in early psychosis, most did not use a standardised experimental design, leaving uncertainties regarding the feasibility of randomising participants and the relevance of certain outcomes to evaluate effectiveness in a subsequent definitive trial.
- Our primary feasibility outcomes (screening and recruitment rates) indicate that our study setup did not provide sufficient incentives and/or infrastructure to ensure consecutive screening and systematic promotion by the case managers and psychiatrists in charge of medical treatment; however, these issues appeared to be unrelated to the application of a randomised design.
- Modifications, including involvement of peers as part of the recruitment strategy, provision of flexible exercise schedules and the option of choosing low intensity/relaxation exercises on days with high symptom burden/anxiety, are required to conduct a large-scale randomised controlled trial and to achieve sustained exercise attendance and adherence.

## Background

Schizophrenia is a severe psychiatric disorder characterised by hallucinations or delusions and experiences that alter perception, thoughts, emotionality and behaviour. The clinical symptoms usually manifest in early adult life [1, 2], and many patients experience persistent difficulties. Previous research indicates that specialised interventions that take place soon after the onset of the first episode of psychosis are associated with reduced symptoms and improved overall functioning [3]. Consequently, specialised early intervention teams constitute standard treatment for first-episode psychosis in many developed countries [3–5]. Concurrent with improved care and outcomes for patients with schizophrenia, prevention of impaired physical health and reduction in premature mortality in patients with schizophrenia are increasingly acknowledged. Patients with schizophrenia have a four-fold risk of metabolic syndrome [6], and almost 1 in 3 of unselected patients with schizophrenia suffer from metabolic syndrome [7]. Accordingly, patients with schizophrenia have a two- to three-fold higher risk of cardiovascular diseases compared to the general population [8], contributing to a premature mortality of 15–20 years observed in people with schizophrenia [9–11] and an increasing mortality gap [12].

An international team of researchers, clinicians and key stakeholders (i.e. The Lancet Psychiatry Commission) recently pointed to physical activity as one key modifiable factor of importance to protecting physical health in people with mental illness [13]. A 2015 review by Firth et al. [14] suggested that exercise can improve cardiometabolic risk factors, functional disability, psychiatric symptoms, co-morbid disorders, and neurocognition in schizophrenia. However, the quality of the included studies was low, and they mainly involved patients with established schizophrenia (i.e. median illness duration was 10 years), leaving limited potential for preventing development of cardiometabolic comorbidity [14, 15].

Treatment of schizophrenia by means of antipsychotic therapy is widely associated with weight gain and metabolic changes that may occur already within weeks after initiation of exposure [16–18]. For this reason, early psychosis could be the optimal phase for introducing exercise to prevent or mitigate comorbid metabolic abnormalities and physical disorders [14]. However, to our knowledge, only few previous studies [19–23] have explored exercise as the primary/dominant intervention module in early psychosis.

Specifically, Fisher et al. recently published the results of a randomised feasibility study investigating exercise quality, engagement and effect of a 12-week intervention involving exercise training (40–60 min 2–3 times a week) in 22 male mental health service users with psychosis (24.8 ± 4.8 years) [22, 23]. While the authors did not observe significant changes in weight or body mass index in either group, the study demonstrated that engaging first-episode psychosis patients in exercise was possible [22, 23]. Moreover, previous research [24] assessing which types of exercise were preferred by patients with early psychosis indicated that gym-based activities, both resistance training and cardio, were substantially more popular than other sporting activities, and that increased fitness/energy, taking your mind off things, and being more confident at the gym were the strongest motivating factors. As such, gym-based exercise that incorporates aerobic and strength training represents a possible novel adjunct clinical pathway to care in young people with early psychosis [13, 25].

With the aim to investigate whether it was possible to recruit and retain patients with early psychosis for a supervised, gym-based exercise training programme, we developed and conducted a feasibility trial called COPUS. Previously published qualitative findings [26] indicated that participants found the programme appealing and valued its ability to create an environment that was equally challenging and caring. The fact that the programme was delivered in a non-clinical setting at a commercial fitness centre enhanced the feeling of being like a normal young adult in a real-world, conventional setting under the supervision of non-health professionals.

In the current study, we aimed to establish screening rate, recruitment rate, retention rate, attendance, and adverse events. Furthermore, we wished to explore heart rate response during the programme, and potential physiological changes when compared to treatment as usual.

## Methods

### Trial design

The study was designed as a mixed-methods, two-arm, randomised feasibility trial with repeated prospective assessments at baseline, after 8 weeks, and after the 16-week follow-up. The study was carried out in accordance with the CONSORT Extension to Pilot and Feasibility Trials, and an additional file provides a completed checklist (See Additional file 1). The results of a qualitative investigation that included participant experiences have been published elsewhere [26]. The ClinicalTrials.gov identifier is NCT03409393. The full trial protocol (in Danish) is available on request by contacting the corresponding author.

### Setting

The study was conducted within specialised multidisciplinary outpatient treatment units, called OPUS teams, offering early intervention treatment to patients in Denmark 18–34 years of age with first-episode psychosis. OPUS is a well-documented intensive specialised treatment modality consisting of three core elements: (1) modified assertive community treatment, (2) family involvement and (3) social skills training. The patient-case manager ratio should not exceed 11:1 [4].

### Population

Inclusion criteria were 18–34 years of age with a recent International Classification of Diseases and Related Health Problems, 10^th^ revision diagnosis of F20–F29 (schizophrenia, schizotypal and delusional disorders and other non-organic psychotic disorders). To ensure stable treatment and to avoid interference with concurrent clinical trials, patients were required to have been enrolled in OPUS for at least 6 months, corresponding to a minimum of 24 consecutive weeks of treatment with antipsychotic medication based on individual clinical needs. If enrolled in OPUS for less than 6 months, evaluation by the treating psychiatrist was required.

### Recruitment

With a recruitment target of 30 patients in 3 months (December 2017 to February 2018), patients were recruited from three OPUS units in Denmark in the greater Copenhagen area. The recruitment period began 6 weeks prior to the baseline test. In order to discuss optimal recruitment procedures, OPUS case managers were invited to a kick-off meeting where it was determined that each case manager would perform an initial screening assessment of patient eligibility and motivation together with the patient. An assessment manual was developed, and one research team member was assigned a desk at the OPUS facility to participate actively during the entire recruitment period. Patients who were deemed eligible were invited to an individual information meeting with a project staff member, at which point they received a detailed oral and written description of the study, including the additional and final screening. After informed written consent was obtained, patients were invited to baseline assessment prior to randomisation.

### Randomisation

Immediately after baseline assessment, included patients were randomly assigned 1:1 to either the exercise intervention group (INT) or a control group (CON). Randomisation was based on a randomisation list generated by an external collaborator in STATA 15.1. Based on this list, 30 numbered, closed and opaque envelopes were stored and managed by an external collaborator different than the one who performed the randomisation. A research assistant who helped with scheduling orally told the study participants on site about the randomisation result. Given the nature of the intervention, it was not possible to blind personnel or participants to the group assignment; however, the data manager/statistical consultant did not participate in the outcome assessment or data entry.

### Intervention

The intervention consisted of 8 weeks of supervised group-based, multifaceted exercise training for 1 h three times a week (twice a week at 11–12 am and once a week at 2–3 pm), with participants recommended to take part in at least two sessions per week. Inspired by CrossFit®, the training sessions comprised warm-up exercises followed by one or two playful, physically demanding games (e.g. dodgeball) and the workout of the day, typically consisting of circuit training with functional movements (resembling activities of daily living) and/or constantly varying movements. This type of functional training was chosen due to its potential to create a sense of community and enjoyment compatible with that presented in sporting activities [27, 28]. Sessions concluded with stretching exercises and, once a week, participants were invited to chat and have refreshments consisting of free fruit and juice. The intensity and complexity of the exercises were increased gradually to prevent injuries, and participants were encouraged to suggest exercises and types of music or specific songs. The sessions, which took place at a fitness centre 3 km away from the OPUS facility, were supervised by a trained physiotherapist and exercise physiologist, supported by undergraduate students from the Department of Nutrition, Exercise and Sports, University of Copenhagen.

To support attendance, short text messages were sent to each participant the day before each training session, encouraging participants to show up. Furthermore, a closed Facebook® group was established for participants to share information on the intervention. In light of research documenting the value of goal setting for behaviour change [29] and performance enhancement [30], not to mention the desire to mark completion of the trial, participants were also given the option of receiving a free ticket to participate in Copenhagen Warrior on 16 June 2018, a 6-km obstacle race with approximately 30 obstacles. The race was promoted as a social event as much as a sporting event, which meant emphasis was also put on the option of participating as a spectator instead of doing the actual race.

### Control group

All participants received treatment as usual. Patients allocated to CON were encouraged to continue normal physical activity. After 8 weeks, i.e. after participation in the 8-week follow-up, participants were offered the intervention, including an invitation to participate in the obstacle race.

### Outcome measures

#### Primary outcomes

Screening rate was defined as the number of patients who had undergone OPUS treatment for a minimum of 6 months divided by the number of patients who were screened for eligibility by OPUS case managers. Recruitment rate was defined as the number of patients screened by OPUS case managers divided by the number of patients who consented to taking part in the study. Retention rate was defined as the number of participants who remained in the study, i.e. the number of participants who did not drop out. Attendance to the intervention was measured by counting how many exercise sessions each participant attended and then dividing it by the total number of exercise sessions. Adverse events were classified according to Good Clinical Practice definitions, i.e. any unfavourable and unintended sign, symptom, or disease temporally associated with the intervention whether or not related to the intervention [31]. Any adverse events were monitored prospectively by the study personnel and immediately communicated to the principal investigator (JM) and subsequently discussed with the clinical medical doctor (BHE) before being recorded as related or unrelated to the study.

#### Secondary outcomes

Heart rate response during training was assessed for the intervention group in the first 8 weeks using ActiGraph wGT3x+ (ACTIGRAPH, Pensacola, Florida, USA) activity monitors. Cardiorespiratory fitness (VO_2max_) was estimated using a direct determination of maximum oxygen uptake following recognised standards [32]. The tests were carried out on a Monark ergometer bike (Monark Exercise, Vansbro, Sweden), and a breath-by-breath respiratory gas analysis (COSMED CPET, Cosmed, Rome, Italy) was used during both tests to take measurements. Body composition, including fat percentage and muscle mass, was assessed using the InBody 570 bioelectrical impedance scale (InBody Co., Ltd., Seoul, Korea). Muscle strength included assessment of a 30-s sit-to-stand chair test, hand grip strength by means of a digital hydraulic dynamometer (NexGen Ergonomics Inc., Quebec, Canada) [33] and measurement of vertical jump height using the OPTOJUMP modular system (MICROGATE, Bolzano-Bozen, Italy) [34]. Balance was measured by means of a single-leg flamingo balance test [35]. Resting heart rate and blood pressure were measured three times at 30-s intervals according to the American Heart Association guidelines for blood pressure measurement [36]. The average of the three measurements was used as the test result.

### Sample size estimation

Since the primary aim of the current study was to establish feasibility, no a priori power calculation was performed to determine statistical power to detect between-group differences.

### Data management and analysis

Study data were collected and managed using Research Electronic Data Capture (REDCap) electronic data capture tools hosted at the Centre for IT, Medical Technology and Telephony Services, Capital Region of Denmark. REDCap is a secure, web-based application designed to support data capture for research studies [37]. The statistical analyses were performed using STATA version 15.1. In accordance with the CONSORT Extension to Pilot and Feasibility Trials [38], analyses were descriptive. Outcomes were assessed using standard methods for rates, proportions, percentages and sample means. Means and 95% confidence intervals (CI) are reported for secondary outcomes at baseline and the 8-week and 16-week follow-ups for each group. Participants were analysed as part of the group they were allocated to regardless of post-randomisation exercise behaviour.

## Results

### Patient characteristics

There were 19 female and six male patients with early psychosis (mean age = 25.5 years, standard deviation (SD) = 4.5, range = 18–35) and a mean body mass index of 25.1 (SD = 5, range = 18–39). Table 1 lists additional characteristics of included patients.

**Table 1:** Demographic and medical characteristics of included patients

Variable	Intervention group (*n* = 13)	Control group (*n* = 12)
Age in years (SD)	23.8 (4.5)	27.5 (4.5)
Females *n* = (%)	9 (69%)	10 (83%)
Weight in kg (SD)	80.3 (18.5)	67.0 (16.1)
Height in cm (SD)	174.4 (8.9)	167.2 (9.0)
Body mass index in kg/m^2^ (SD)	26.4 (5.7)	23.7 (21.3)
Diagnosis retrieved from patient medical record^a^		
F20 schizophrenia	7	5
F21 schizotypal	5	5
F29 non-organic psychoses	1	2
Self-reported physical activity^b^		
Almost completely inactive	2	3
Moderate (2–4 h a week)	6	5
Moderate (4 h a week)	4	3
Strenuous (> 4 h a week)	1	1
Regular smoking		
No	9	8
Yes	4	4
Cannabis use		
Never tried	8	8
Tried few times	4	2
Regularly use	1	2

Mean; (SD) standard deviation; ^a^assessed according to International Statistical Classification of Diseases and Related Health Problems, 10^th^ Revision; ^b^assessed according to Saltin-Grimby Physical Activity Level Scale [39]

### Primary outcomes

**Fig. 1:**
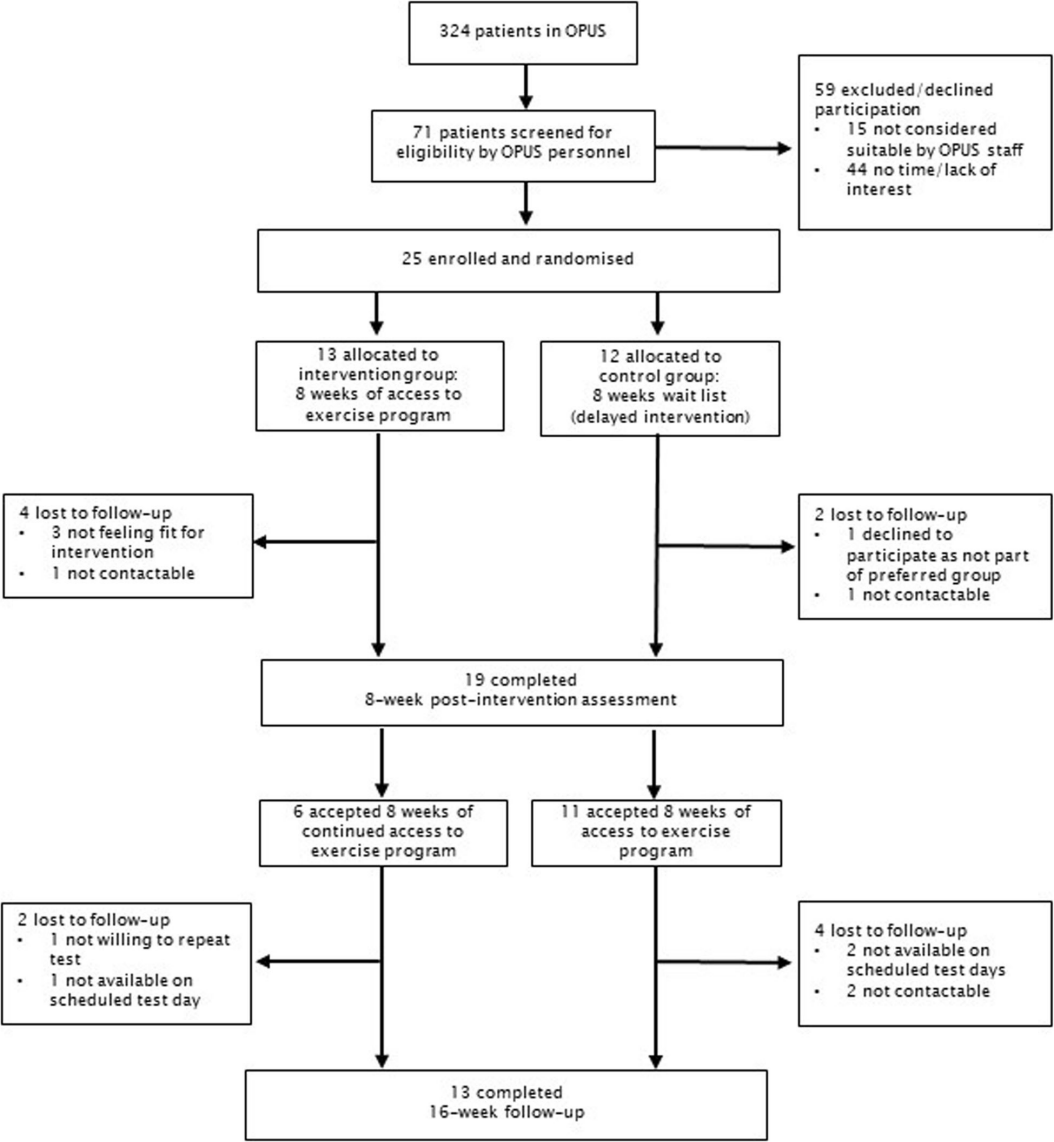
Participant flow diagram for trial

The screening rate was 27% and the recruitment rate was 35%. Specifically, 21 out of 30 OPUS case managers (70% of total staff) screened 86 out of the 324 patients receiving OPUS treatment. Out of the 86 patients who were screened, 49 (56%) were screened by five case managers (17% of total staff). Fifteen (17%) of the 86 screened patients were subsequently excluded, mainly due to time constraints or disease-related causes (symptom burden). In total, 71 out of the 86 (83%) patients were deemed eligible to participate, of which 25 (35%) agreed to inclusion and were randomly assigned to INT (*n* = 13) or CON (*n* = 12). Five participants (38%) chose to discontinue before beginning the intervention or were lost to follow-up prior to completing the intervention. Retention at the 8-week follow-up was 76%, and at 16-weeks it was 52%. Figure 1 illustrates participant flow during the study.

Patients in INT who completed pre- and 8-week post-intervention assessments attended a mean of nine out of the 22 training sessions, corresponding to an attendance rate of 43% (min. 9%, max. 96%). Six (46%) patients allocated to INT and 11 (92%) to CON agreed to exercise from week 8 to week 16 (attendance rate 35% for INT and 44% for CON). Ten patients (5 from INT, 5 from CON) completed the obstacle race, which took place 2 weeks after study completion. During the assessment periods, one adverse event was registered and involved compulsory admission to hospital. The patient was assessed by the treating psychiatrist, who concluded that the event was unrelated to the intervention, and the patient was allowed to continue participation in the exercise training sessions. No other adverse events were reported during the study, although some mentioned transient, mild muscle soreness as a direct result of exercise training.

### Secondary outcomes

The exercise training programme was performed at moderate intensity (65–85% HR_max_) 41% of the time and at high intensity (85–100% HR_max_) 19% of the time. No changes were detected in either groups in cardiorespiratory fitness. However, significant improvements were observed in INT in sit-to-stand from baseline to the 8-week follow-up (mean difference 2.8 repetitions, 95% CI 0.4 to 5.1) and in jump height from 8 to 16 weeks (mean difference 1.6 cm, 95% CI 0.2 to 2.9). In CON, significant improvements were observed in sit-to-stand from baseline to the 8-week follow-up (mean difference 1 repetition, 95% CI 0.3 to 1.7) and from 8 to 16 weeks (mean difference 1.7 repetitions, 95% CI 0.7 to 2.7). Moreover, significant improvements were seen from week 8 to week 16 in CON in muscle mass (mean difference 0.8 kg, 95% CI 0.3 to 1.4) and in jump height (mean difference 1.3 cm, 95% CI 0.1 to 2.5). Table 2 lists means and 95% CIs on cardiorespiratory fitness and secondary outcomes measures.

**Table 2:** Means and 95% confidence interval on cardiorespiratory fitness and secondary outcome measures

	VO_**2**_max (mL/min)	Resting heart rate	Diastolic blood pressure	Systolic blood pressure	Fat (%)	Muscle mass (kg)	Sit to stand 30 (repetitions)	Grip strength (kg)	Jump height (cm)	Balance test (falls, ***n***)
**Intervention group**										
**Baseline**										
Mean	2823	75.2	77.1	118.7	29.1	31.2	18.4	35.2	20.4	16.9
95% CI	2328 to 3318	66.6 to 83.9	72.9 to 81.3	112.8 to 124.6	22.7 to 35.5	26.3 to 31.7	16.3 to 20.4	30.2 to 40.1	15.6 to 25.3	12.9 to 20.9
**8 weeks**										
Mean	2775	81.4	80.5	117.4	26.3	30.6	22.4	37.1	22.5	14.6
95% CI	1755 to 3794	64.0 to 98.7	72.2 to 88.8	105.7 to 129.1	15.3 to 37.2	24.6 to 36.6	18.6 to 26.2	27.5 to 46.8	16.7 to 28.4	10.6 to 18.6
Change between baseline and 8 weeks										
Mean change	183	8.1	1.8	-3.6	1.2	-0.5	2.8	1.4	-0.4	-2.0
95% CI	− 250 to 617	− 10.8 to 27.0	− 3.3 to 6.8	− 12.0 to 4.7	− 1.2 to 3.7	− 2.4 to 1.4	0.4 to 5.1	− 2.3 to 5.0	− 2.3 to 1.5	− 6.5 to 2.5
**16 weeks**										
Mean	3110	80.0	74.2	123.5	22.8	29.2	22.5	32.8	25.0	12.3
95% CI	1550 to 4670	68.9 to 91.1	64.0 to 84.3	112.9 to 134.1	9.6 to 36.1	20.8 to 37.6	19.5 to 25.5	24.7 to 41.0	17.1 to 32.8	7.0 to 17.7
Change between 8 weeks and 16 weeks										
Mean change	232	2.5	− 2.5	10.2	− 0.4	0.5	− 0.2	− 1.2	1.6	− 0.8
95% CI	− 1531 to 1996	− 17.0 to 22.0	− 7.6 to 2.6	− 2.2 to 22.6	− 2.8 to 2.0	− 0.2 to 1.2	− 3.9 to 3.6	− 6.8 to 4.4	0.2 to 2.9	− 5.2 to 3.5
**Wait**-**list control group**										
**Baseline**										
Mean	2315	78.8	78.4	113.9	27.3	26.6	16.3	32.8	19.0	15.7
95% CI	1790 to 2839	68.3 to 89.2	74.2 to 82.7	107.7 to 120.1	22.7 to 31.8	23.0 to 30.3	14.4 to 18.1	29.0 to 36.5	15.4 to 22.7	11.6 to 19.7
**8 weeks**										
Mean	2399	81.4	77.8	113.1	26.0	26.5	17.3	34.6	19.3	15.0
95% CI	1719 to 3080	70.6 to 92.1	73.3 to 82.4	106.1 to 120.0	20.5 to 31.4	22.1 31.0	15.1 to 19.4	29.3 to 40.0	15.8 to 22.9	11.5 to 18.5
Change between baseline and 8 weeks										
Mean change	11.0	2.1	− 0.8	− 1.5	− 0.5	− 0.2	1.0	1.6	0.5	− 0.6
95% CI	− 106 to 127	− 8.0 to 12.2	− 4.7 to 3.0	− 7.2 to 4.1	− 2.7 to 1.7	− 0.9 to 0.6	0.3 to 1.7	− 0.6 to 3.9	− 1.0 to 1.1	− 4.5 to 3.2
**16 weeks**										
Mean	2234	73.9	69.3	115.7	25.0	25.5	20.0	33.1	21.6	11.3
95% CI	1562 to 2907	61.6 to 86.1	63.1 to 75.5	109.0 to 122.4	15.6 to 34.5	21.9 29.2	17.0 to 23.0	27.4 to 38.9	16.0 to 27.3	8.4 to 14.1
Change between 8 weeks and 16 weeks										
Mean change	− 4	− 4.1	− 8.3	5.7	− 0.6	0.8	1.7	− 0.7	1.3	− 1.9
95% CI	− 328 to 320	− 13.0 to 4.7	− 13.1 to− 3.5	− 1.0 to 12.4	− 4.0 to 3.9	0.3 to 1.4	0.7 to 2.7	− 2.4 to 0.9	0.1 to 2.5	− 4.9 to 1.2

## Discussion

Despite early psychosis being proposed as the optimal phase for using exercise [14], the current study is among the first randomised controlled trials to assess the feasibility of an exercise training programme compared to usual care in patients with first-episode psychosis undergoing specialised early intervention.

Our primary feasibility outcomes indicate that our study setup did not provide the case managers and psychiatrists in charge of medical treatment with sufficient incentives and/or infrastructure to ensure consecutive screening and systematic promotion of recruitment. Despite a successful kick-off meeting, positive initial feedback from OPUS case managers on their central role in screening and recruitment and continuous encouragement and practical help from the research team, we did not reach our recruitment target of 30 patients. More than half of screened patients were screened by less than 20% of case managers. The OPUS caseload is 1:11 (i.e. one case manager for 11 patients), but only five case managers (17% of staff) screened more than seven patients, whereas 14 (47% of staff) screened three patients each or less. While staff motivation for active involvement in recruitment of patients remains unexplored in the current study, a recent qualitative study suggested an uncertainty among nurses concerning the benefits of physical activity as complementary treatment in patients with schizophrenia, indicating that hidden resistance exists in terms of the concept overall [40]. Moreover, a recent study [41] indicated that exposing mental health staff to lifestyle interventions prior to targeting patients is critical to instigating culture change and improving patient outcomes. As such, closer collaboration with staff, including shared ownership and potentially staff-focused interventions, appears warranted to support recruitment of study participants in future exercise trials. Consequently, subsequent to trial completion, we invited participants and OPUS staff to an evaluation meeting, which resulted in the following additional suggestions for improved screening, recruitment and retention rates in future trials: provide a trial exercise session to staff and potential participants; involve peers as part of the recruitment strategy; focus on implementation of strategies to maintain exercise post-intervention; have flexible exercise schedules; and provide the option of choosing low intensity/relaxation exercises on days with high symptom burden/anxiety. Interestingly, these suggestions also reflect known barriers for participation in exercise for people with severe mental illness, e.g. stress, fatigue and lack of social support [25]. Hence, population-specific exercise barriers need to be considered as contributing factors in explaining the relatively low attendance rate of 43% in the current study. In comparison, Fisher et al. [23] reported an attendance rate of 83% for a 12-week intervention involving free-of-choice exercise training (2–3 times a week for 40–60 min) in male mental health service users with first-episode psychosis (24.8 ± 4.8 years). While it is likely that providing participants with a choice of different activities may have increased attendance, the considerable discrepancy in reported attendance rates between our study and Fisher et al.’s [23] must be interpreted with caution due to a lack of consensus on calculating/defining attendance, the small number of randomised participants (*n* = 27 and *n* = 22, respectively) and the substantial attrition in both studies (24% and 32%, respectively).

In this trial, secondary outcomes were selected based on the assumption that the physical health of participants would undergo measurable improvements attributable to our intervention. However, because the current trial was not designed or statistically powered to test for differences between treatment arms, no between-group analyses were performed. The statistically significant within-group changes were nonetheless observed in sit-to-stand in INT from 0 to 8 weeks and in CON from 8 to 16 weeks, which indicates that the intervention may have had an impact on functional capacity, whereas no changes were observed in fitness, which may be related to the limited training frequency and the fact that only 19% of the intervention was performed at high intensity. Notably, 80% of participants reported being at least moderately physically active (minimum 2 h per week) at baseline, suggesting that those patients already interesting in exercise were more likely to participate, making it less likely that marked increases in fitness could be achieved. Also, because participants had been on antipsychotic treatment for a minimum of 24 weeks ahead of enrolment, it is possible that metabolic changes had already occurred, and that earlier initiation of exercise (i.e. before or concurrent with initiation of antipsychotic medication) would have been preferable. For example, attenuation of expected decreased fitness and prevention of weight gain, for which patients with first-episode psychosis are particularly susceptible, may be [42] a more realistic goal than improved physiological and functional outcomes. Moreover, it is worth considering whether simultaneously targeting multiple lifestyle factors (e.g. poor diet and smoking) instead of focusing on one behavioural modification, i.e. increasing physical exercise, may be more appropriate, as suggested by The Lancet Psychiatry Commission [13]. Yet, the perceived changes in some outcomes (and the lack of changes in other others) in the current study should be interpreted with great caution and should not be ascribed a positive physiological effect (or the opposite). Also, the relatively small number of randomised patients and the large variation in outcomes make it impossible to derive an obvious candidate as the primary outcome for a large-scale randomised controlled trial based on the results from this feasibility trial.

However, while the present study is among the first randomised trials evaluating feasibility of exercise training in first-episode psychosis, several reviews and recent meta-reviews have investigated the effects of physical activity and/or exercise-based interventions in people with severe mental illness, including schizophrenia-spectrum disorders [13, 43]. This body of evidence suggests that exercise can result in significant benefits across multiple cardiometabolic outcomes (e.g. fasting glucose and waist circumference), as well as improve clinical symptoms (including negative symptoms), quality of life, global functioning and depressive symptoms in people with schizophrenia [44–47]. In addition, effect on cognition has not been demonstrated but may be present for low-intensity exercise (e.g. yoga) [45, 48]. Subjective outcomes were not included in the current trial due to an expected lack of statistical power; however, it should be considered whether functional measures and measures of psychopathology may be superior or equally relevant to physiological endpoints in a future large-scale trial. An important recently published study identified social and non-social cognition, avolition and positive symptoms as the main factors associated with real-life functioning in people with schizophrenia, suggesting that interventions targeting and promoting cognition and independent living may be especially relevant in the management and recovery of schizophrenia [49]. In this regard, it is interesting to note that our recently published qualitative sub-study [26] indicated that participants in the present trial appreciated the intervention due to its potential to support the recovery process by creating a socially inclusive environment that was as challenging as it was caring [26]. Participants especially valued the opportunity to take part in an activity delivered in a non-patient environment, and some described the intervention as providing a welcome distraction from symptoms, while some reported improved sleep [26]. As such, measuring recovery, sleep quality, internalised stigma of mental illness and/or loneliness may be especially relevant to include in future research. Moreover, prior research indicates that incorporating self-efficacy building techniques facilitates health behaviour change in people with severe mental illness [50] and that implementation of self-monitoring, goal setting and feedback may thus be imperative to secure long-term exercise motivation.

## Conclusions

In conclusion, the current study elucidated operational factors in relation to feasibility in terms of applying a randomised controlled trial design to provide a rigorous evaluation of the effects of exercise training in patients with first-episode psychosis. These aspects have been taken into consideration in the design of a future trial that will involve a multi-centre, pragmatic clinical trial intended to examine the effectiveness of exercise training in a non-clinical setting as potentially promoting health. As future endpoints, more recovery-related measures, rather than a strict focus on objective physiological measures, may prove relevant.

## Supplementary Information

**Additional file 1.** CONSORT checklist.

## Data Availability

The datasets used and/or analysed during the current study are available from the corresponding author on reasonable request.

## Abbreviations

CI: Confidence interval
CON: Control group
INT: Intervention group
REDCap: Research Electronic Data Capture
SD: Standard deviation
